# Self-Humidifying and Super-Protonic Conductivity of SPEEK-Based Composite Proton Exchange Membranes Incorporated by Functionalized MXene and Modified TiO_2_ Nanofillers

**DOI:** 10.3390/nano16080446

**Published:** 2026-04-08

**Authors:** Manting Huang, Ai Song, Xingliu Ben, Weijia Ji, Yuxuan Pan, Huaxin Rao

**Affiliations:** College of Chemistry and Materials Science, Jinan University, Guangzhou 510632, China; manting0223@163.com (M.H.); song102525@163.com (A.S.); 13621537426@163.com (X.B.); 13768059408@163.com (W.J.); billypoon1021@163.com (Y.P.)

**Keywords:** proton exchange membrane fuel cells, PEEK, MXene, TiO_2_ nanoparticle, nanofillers, composite membrane, proton conductivity, self-humidifying, synergic action

## Abstract

MXene, as a suitable and alternative 2D nanofiller incorporated into a proton exchange membrane (PEM), has recently received considerable attention because of desired mechanical stability, promising conductivity, and active surface functional groups. However, agglomeration or sedimentation in PEMs, as well as the water retention capacity under low humidity of MXene, are limiting factors in the field of PEMs. In this paper, modified MXene and TiO_2_ nanoparticles used as functional nanofillers were incorporated into sulfonated poly (ether ether ketone) (SPEEK) to prepare novel SPEEK-based composite PEMs. The effects of the nanofiller contents on self-humidifying and protonic conductivity of the composite PEMs were also investigated under different temperatures. When the contents of functionalized MXene and modified TiO_2_ are 5 wt.%, proton conductivity, water uptake and methanol permeability of the composite PEMs can be up to 0.143 S/cm, 60% and 2.27 × 10^−7^ cm^2^/s, respectively, which represent increases of about 192%, about 38% and a decrease of 47%, respectively, compared with that of primary SPEEK PEM. Under the synergistic action of functionalized MXene providing a higher number of exchangeable proton sites, modified TiO_2_ with inherent hydrophilicity enhancing water retention and Pt providing catalytic sites for the H_2_/O_2_ reaction to generate water in situ, the self-humidifying capability and proton conductivity of the composite PEMs were improved significantly.

## 1. Introduction

In order to address energy security and environmental issues, it is particularly important to develop green renewable energy sources and new technologies that are environmentally friendly and highly efficient [1]. Proton exchange membrane fuel cells (PEMFCs) have received considerable attention due to their environmental friendliness and relatively low cost. The proton exchange membrane (PEM) is considered to be the core component of PEMFCs and needs to have low fuel permeability, high proton conductivity, good thermal stability, excellent antioxidant ability and excellent mechanical properties. Perfluorosulfonic acid membranes (such as Nafion) are the most widely used membranes on the market and in the field of PEM research due to their excellent chemical stability and mechanical properties [2,3,4,5,6]. However, some limitations, such as high manufacturing costs [7], high fuel permeability and low proton conductivity at temperatures exceeding 80 °C inhibit their development in fuel cell applications. Therefore, inexpensive, easily available and low-permeability aromatic polymers have become promising alternative PEMs to perfluorinated PEMs.

Among the aromatic polymers, polyetheretherketone (PEEK) has been proven to be an economical alternative PEM due to its good thermal stability, chemical stability and high proton conductivity at an appropriate degree of sulfonation (DS) [8]. However, PEEK-based PEMs have some disadvantages, such as excessive swelling and poor mechanical properties at high DS. In view of the shortcomings of perfluorosulfonic acid membranes and of non-fluorinated aromatic polymer membranes in a high-temperature and low-humidity working environment, it is an effective and practical method to prepare low-cost and high-performance inorganic–organic composite PEMs. As systematically reviewed by Li, recent advances in sulfonated PEEK (SPEEK) composite membranes highlight their potential for enhancing proton conductivity and reducing fuel permeability in PEMFCs, making them a promising research direction [9]. Over the last few decades, significant efforts have been dedicated to exploring PEEK-based composite PEMs by introducing some inorganic components (such as metal oxides, clays and zeolites) in the SPEEK polymer matrix. For example, SiO_2_ and TiO_2_ inorganic nanoparticles can provide certain water adsorption sites for the PEM due to their hydrophilicity, which effectively improves the water uptake of the PEM [10,11,12,13,14]. The introduction of the inorganic components can enhance the water retention and thermal properties of PEEK-based composite PEMs to a certain extent, but the proton conductivity of the composite PEMs is not significantly increased, and the leaching of inorganic fillers from the membranes is a major drawback of these types of composite PEMs. In addition to traditional metal oxide fillers, recent studies have shown that tungsten oxide (WO_3_) nanoparticles can significantly improve the proton conductivity of SPEEK-based membranes [15]. However, for PEMFC operation under elevated temperatures and low humidity, relying solely on a single type of metal oxide filler—whether conventional (SiO_2_, TiO_2_) or non-traditional (WO_3_)—often fails to simultaneously meet the requirements of high proton conductivity, adequate water retention and long-term stability due to issues such as insufficient water retention or poor compatibility with the SPEEK matrix.

Beyond the aforementioned metal oxides and clays, recent years have witnessed extensive research on incorporating advanced nanofillers such as graphene oxide (GO) [16] and other two-dimensional (2D) materials (e.g., MoS_2_, g-C_3_N_4_) [17] into PEMs, as well as hybrid filler systems that combine multiple functionalities. Amino-functionalized carbon nanotubes have also been reported to effectively improve the performance of SPEEK composite membranes [18]. These emerging fillers can provide additional proton-conducting sites, enhance mechanical reinforcement or introduce self-humidifying capabilities, thereby offering new strategies to overcome the limitations of conventional composites. Accordingly, rational design and incorporation of functional inorganic nanofillers have become an effective strategy to further upgrade the performance of PEMs.

MXene, as a suitable and alternative 2D nanofiller incorporated into PEMs, has recently received considerable attention because of its desired mechanical stability, promising conductivity and active surface functional groups [19,20,21]. However, its agglomeration and sedimentation in PEMs, as well as its insufficient water retention capacity under low humidity, restrict its practical application in PEMs.

Based on the above considerations, the aim of this study is to develop novel SPEEK-based composite PEMs using functionalized MXene and modified TiO_2_ as functional nanofillers in order to increase proton conductivity and self-humidification under different temperatures. First, Pt-TiO_2_ nanoparticles were prepared by a redox method, and the MXene material was treated with alkali to introduce Na^+^ into the gaps between Ti_3_C_2_ layers. Subsequently, the benzenesulfonic acid groups released after the hydrolysis of p-aminobenzenesulfonic acid were grafted onto the surface of the MXene material by diazotization reaction to prepare functionalized MXene (f-MXene) with sulfonic acid groups. Using SPEEK as the composite polymer membrane matrix, a series of SPEEK/MXene/Pt-TiO_2_ composite PEMs with different nanofiller contents were prepared by the solution-casting technique, and the effects of Pt-TiO_2_ and MXene contents on the performance of PEMs were also investigated.

## 2. Materials and Methods

### 2.1. Materials

MXene (Ti_3_C_2_, 100–200 nm) was purchased from Jiangsu XFNANO Materials Technology Co., Ltd. (Nanjing, China) Sulfanilic acid and PEEK were obtained from Shanghai Macklin Biochemical Co., Ltd. (Shanghai, China) Sodium nitrite was supplied by Shanghai Jizhi Biochemical Co., Ltd. (Shanghai, China) N,N-Dimethylacetamide (DMAc), NaBH_4_, H_2_PtCl_6_, concentrated sulfuric acid and hydrochloric acid were provided by Guangzhou Chemical Reagent Factory (Guangzhou, China). Chloroplatinic acid and titanium dioxide (TiO_2_, 40 nm) were purchased from Aladdin (Shanghai, China). 3-Aminopropyltriethoxysilane (KH-550) was purchased from Shandong Yousuo Chemical Technology Co., Ltd. (Linyi, China) Anhydrous ethanol was obtained from Shanghai Titan Technology Co., Ltd. (Shanghai, China). Phenolphthalein was obtained from Tianjin Fine Chemical Technology Development Center (Tianjin, China).

### 2.2. Modification of TiO_2_

TiO_2_ (0.1127 g) was dispersed in 100 mL anhydrous ethanol under ultrasonication for 1 h. An aqueous solution of H_2_PtCl_6_ (0.155 g, 0.008 mol/L) was added into the TiO_2_ suspension, and then NaBH_4_ (0.03 g, 0.03 mol/L), used as the reducing agent, was added dropwise to the mixture. The mixture was stirred vigorously until it turned black. Then the product was separated by centrifugation and washed with water and anhydrous ethanol twice, respectively. The final product was dried in an oven at 80 °C for 12 h to obtain Pt-TiO_2_ nanoparticles.

The resulting Pt-TiO_2_ nanoparticles were added into anhydrous ethanol, and the pH value was adjusted to 10. The mixture was stirred magnetically for 30 min, followed by ultrasonic dispersion for 30 min. The dispersion containing KH-550 (with a mass fraction of 15% relative to Pt-TiO_2_) was placed in a three-necked flask and then reacted under reflux and stirring at 60 °C for 1 h. Finally, the product was centrifuged and redispersed in 100 mL anhydrous ethanol under ultrasonication. The resulting product was dried in an oven at 80 °C for 12 h to obtain Pt-TiO_2_ nanoparticles modified by silane coupling agent KH-550.

### 2.3. Preparation of SPEEK

The preparation of SPEEK has been reported in the literature [22,23]. A homogeneous solution was formed by mixing dried PEEK particles (2.5 g) with concentrated sulfuric acid (50 mL) under stirring, and the mixture was reacted in a water bath for 4 h. The resulting product was transferred to a separating funnel, and the post-reaction solution was added dropwise into an ice–water mixture. Finally, the resulting SPEEK precipitate was filtered, washed with deionized water until neutral and then dried in a vacuum oven at 80 °C for 12 h to obtain pink spherical SPEEK particles with a DS of about 60%.

### 2.4. Preparation of f-MXene

Preparation of the functionalized MXene was carried out using the in situ generated diazonium salt method. Multilayered MXene (0.01 g) and 1 mol/L sodium hydroxide solution (50 mL) were mixed and sonicated for 1 h. The mixture was washed with deionized water and then centrifuged. The resulting precipitated particles were uniformly dispersed in deionized water (10 mL) to prepare Na^+^ intercalated MXene.

The Na^+^-intercalated MXene suspension was added to a three-necked flask. P-Aminobenzenesulfonic acid (0.46 g) was dispersed in deionized water (10 mL) and then added to the three-necked flask. A 1 mol/L HCl solution was pre-cooled and then added to the flask. The mixture was stirred and reacted in an ice bath for 15 min. Subsequently, NaNO_2_ (0.18 g) was dispersed in deionized water (2 mL) and then added to the three-necked flask. Finally, the mixture was stirred and reacted in an ice bath for 2 h. The product was filtered through a 0.45 µm filter membrane and washed with deionized water, methanol, dimethylformamide, and acetone sequentially to remove residues. The resulting product was dried in a vacuum oven at 60 °C for 12 h to obtain f-MXene.

### 2.5. Preparation of SPEEK/MXene/Pt-TiO_2_ Composite PEM

SPEEK (0.2 g) was dissolved in DMAc (1.8 g) under stirring at room temperature for 12 h. A total of 0.01 g of f-MXene was dispersed in the SPEEK solution and sonicated for 1 h to form a SPEEK/MXene suspension. Subsequently, a SPEEK/MXene composite membrane (denoted as SPEEK/MXene-5) was prepared by the solution-casting method, followed by drying in a vacuum oven at 60 °C for 24 h.

**Scheme 1:**
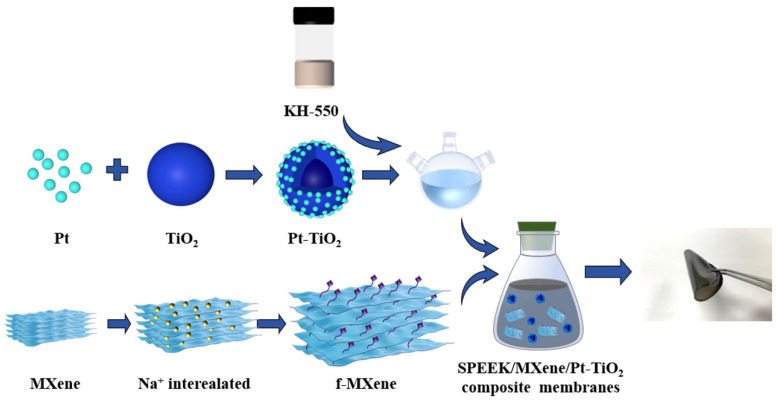
Preparation process of SPEEK/MXene/Pt-TiO_2_-x composite PEMs.

In addition, an amount of modified Pt-TiO_2_ nanoparticles was gradually added into the SPEEK/MXene solution under constant stirring, followed by ultrasonic mixing at room temperature for 12 h to form a SPEEK/MXene/Pt-TiO_2_ suspension. Subsequently, SPEEK/MXene/Pt-TiO_2_ composite membranes were prepared by the solution-casting method and dried in a vacuum oven at 60 °C for 24 h. The resulting composite membranes were denoted as SPEEK/MXene/Pt-TiO_2_-x, where x refers to the content of Pt-TiO_2_ in the composite membrane. SPEEK/MXene/Pt-TiO_2_-2.5, SPEEK/MXene/Pt-TiO_2_-5, SPEEK/MXene/Pt-TiO_2_-7.5 and SPEEK/MXene/Pt-TiO_2_-10 composite PEMs were prepared according to the above membrane-forming conditions, respectively. The preparation process of SPEEK/MXene/Pt-TiO_2_-x composite PEMs is illustrated in Scheme 1.

### 2.6. Characterization and Performance Measurement

#### 2.6.1. Characterization of Modified Pt-TiO_2_

The crystal patterns of TiO_2_ and Pt-TiO_2_ were analyzed by using an X-ray diffractometer (XRD, MiniFlex, Nippon Rigaku, Tokyo, Japan). The scanning angle of the X-ray diffractometer was set in the range of 5–50°, and the scanning speed was set to 4°/min.

The elemental composition of TiO_2_ and Pt-TiO_2_ was analyzed using an X-ray photoelectron spectrometer (XPS, Thermo K alpha^+^, Thermo Fisher Scientific, East Grinstead, UK). The energy resolution of the instrument is ≤0.5 eV, and the X-ray source is an Al Kα target.

The dispersibility of the modified Pt-TiO_2_ nanoparticles was analyzed using an Omni NanoSizer and a Zeta potential analyzer produced by Malvern Instruments (Malvern, UK).

#### 2.6.2. Characterization of SPEEK

The chemical structures of PEEK and SPEEK were tested using a Fourier Transform Infrared (FTIR) Spectrometer. The instrument was the Spectrum Two model from PerkinElmer (Waltham, MA, USA) with a scanning wavelength range of 4000 to 400 cm^−1^.

To determine the DS of SPEEK polymer, a hydrogen nuclear magnetic resonance analysis (^1^HNMR) was performed using a nuclear magnetic resonance spectrometer produced by Bruker Switzerland AG (Fällanden, Switzerland), model AVANCE NEO Ascend 400. Deuterated dimethyl sulfoxide (DMSO-d6) was used as the solvent for each analysis.

#### 2.6.3. Characterization of f-MXene

The structural changes [24] of MXene were measured using a Miniflex 600 X-ray diffractometer from Rigaku Corporation (Tokyo, Japan). The scanning angle of the X-ray diffractometer was set in the range of 5–50, and the scanning speed was set to 4°/min.

The surface morphology of MXene and f-MXene was characterized using a field emission scanning electron microscope (FESEM) model ULTRA 55 manufactured by ZEISS, Oberkochen, Germany, with a Schottky thermal field emission gun.

The X-ray photoelectron spectroscopy (XPS) analysis was conducted using a Thermo K-alpha^+^ X-ray photoelectron spectrometer from Thermo Fisher Scientific, UK.

#### 2.6.4. Measurement of Ion Exchange Capacity (IEC)

IEC was calculated by the standard acid–base titration process. Primary SPEEK membranes, SPEEK/MXene composite membranes and SPEEK/MXene/Pt-TiO_2_ composite membranes with different contents were cut into certain sizes and weighed, respectively. The mass of each membrane was recorded and denoted as *Wd*. The membranes were placed in a vacuum oven at 60 °C for 24 h. After drying, the membranes were placed in 6 conical flasks containing equal amounts of 1 M NaCl solution and left to stand for more than 24 h to ensure that Na^+^ completely replaced H^+^. The membrane samples were taken out of the conical flasks and repeatedly washed with deionized water. The washed solution was collected in the NaCl solution in which the membrane samples were soaked. Finally, phenolphthalein indicator was added to the conical flasks and acid–base titration was performed with 0.01 M *NaOH* solution. The experiment was repeated three times. The IEC of the membrane samples was calculated according to Formula (1).

(1) $$IEC=\frac{C_{NaOH}\times V_{NaOH}}{W_{d}}\times 100\%$$

where *W_d_* is the mass of the dry membrane (g), *V_NaOH_* is the volume of the *NaOH* solution consumed during titration (mL), and *C_NaOH_* is the concentration of the *NaOH* solution (mol/L).

#### 2.6.5. Measurement of Proton Conductivity

The electrochemical workstation model CHI760e manufactured by Shanghai Chenhua Instrument (Shanghai, China) was used to measure the proton conductivity of the membrane by measuring impedance under humid conditions (100% humidity). The experimental conditions included a perturbation voltage of 10 mV and a testing frequency range of 1–10^5^ Hz. The primary SPEEK membrane, SPEEK/MXene and SPEEK/MXene/Pt-TiO_2_ composite membrane were cut into a shape of 20 mm × 10 mm. Subsequently, the membrane samples were immersed respectively in deionized water for 24 h to ensure that the samples were tested under 100% humidity conditions. The membrane thickness was initially recorded, and the membranes were connected with Pt electrode pieces for proton exchange. The water bath temperature was set, and different compositions of composite membranes along with the primary SPEEK membrane were placed in the water bath for testing. The impedance values *R* of the PEM samples at temperatures of 25 °C, 50 °C and 75 °C were measured. Subsequently, the proton conductivity (σ) of the PEMs at different temperatures were calculated using Formula (2).

(2) $$\sigma =\frac{L}{R\times A}$$

where σ (S/cm) is the proton conductivity of the membrane, *L* (cm) refers to the effective distance of the membrane sample between the two platinum electrodes. *R* (Ω) is the impedance value obtained under EIS for different content membrane sample, and *A* (cm^2^) is the effective surface area of the membrane sample.

#### 2.6.6. Measurement of Water Uptake (WU) and Swelling Ratio (SR)

WU of the membranes in this study was determined by the difference in mass between the dry and wet states of the composite membranes. A certain mass of primary SPEEK membrane, SPEEK/MXene membrane and SPEEK/MXene/Pt-TiO_2_ composite membrane were cut and placed in 6 volumetric flasks respectively. Deionized water of the same mass was added to each flask, which was then sealed and placed in ovens set at 25 °C, 50 °C and 75 °C. Three parallel control groups were included in the experiment. After 24 h of static storage, the membranes were removed, excess surface water was wiped off with absorbent paper, and the mass of the membranes in the wet state (*W_w_*) was recorded. Subsequently, the wet membranes were dried in a vacuum oven at 60 °C for 12 h, and the mass of the membranes in the dry state (*W_d_*) was measured. The *WU* of the membranes was calculated according to Formula (3).

(3) $$WU=\frac{W_{w}-W_{d}}{W_{d}}\times 100\%$$

where *W_d_* is the mass of the dry membrane, and *W_w_* is the mass of the wet membrane.

The primary SPEEK membrane, the SPEEK/MXene membrane and the SPEEK/MXene/Pt-TiO_2_ composite membrane were dried at constant temperature for 24 h. The samples were cut into squares measuring 10 mm × 10 mm, and the surface area of the samples in the dry state was measured and denoted as *S_d_*. These squares were then placed in six volumetric flasks, and deionized water of the same mass was added to each flask. The flasks were sealed and placed in ovens set at 25 °C, 50 °C and 75 °C. Three sets of parallel control groups were included in the experiment. After 24 h of static storage, the membranes were removed, and excess surface water was wiped off with absorbent paper. The surface area of the wet membranes was measured and denoted as *S_w_*. The *SR* of the primary SPEEK membrane, SPEEK/MXene composite membrane and SPEEK/MXene/Pt-TiO_2_ composite membrane can be calculated using Formula (4).

(4) $$SR=\frac{S_{w}-S_{d}}{S_{d}}\times 100\%$$

where *S_w_* is the surface area of the membrane in the wet state (cm^2^), and *S_d_* is the surface area of the membrane in the dry state (cm^2^).

#### 2.6.7. Measurement of Thermal Stability, Mechanical Properties and Cross-Sectional Characterization

The thermal properties of the composite membranes were evaluated by thermogravimetric analysis (TGA, TGA2, Netzsch, Selb, Germany). The temperature was scanned from 50 °C to 800 °C at a rate of 10 °C/min in a nitrogen atmosphere.

The mechanical properties of the composite membrane were measured using Shimadzu AGS-X of Kyoto City, Japan. The tensile rate was 50 mm/min, and every sample was tested three times under room temperature.

A scanning electron microscope (SEM, EVO-MA15, Germany Zeiss Company, Oberkochen, Germany) was used to observe the cross-sectional morphology of the composite membranes.

#### 2.6.8. Measurement of Methanol Permeability

The methanol permeability of PEM was characterized by diffusion cell measurements [25], where the methanol permeability was represented by the amount of methanol passing through PEM within a certain time period. Each concentration of methanol solution corresponded to a specific refractive index. In this experiment, the refractive indices of methanol solutions at various concentrations were first measured using an Abbe refractometer to infer the relationship between methanol solution concentration and refractive index. Through calculation and analysis, the relationship between the molar concentration (c) of methanol solutions and the refractive index (*n*) was found to follow Formula (5).

(5) c=1554.32 n−2070.64

First, the primary SPEEK membrane and the composite membrane were immersed in deionized water for 24 h to ensure that the membrane samples were in a wet state for the methanol permeability test. All tests were performed at 30 ± 1 °C. The membrane samples of each content were cut into circular shapes slightly larger than the center of the diffusion cell, and the thickness (*L*) of the membrane was measured. The membrane samples were placed inside the diffusion cell and clamped on both sides of the diffusion cell to secure them in place. On one side, 2 mol/L methanol solution (20 mL) was added, and this side was labeled Chamber A. An equal amount of deionized water was then added to the other side, designated as Chamber B. The diffusion cell was sealed on both sides with cling film, then placed on a magnetic stirrer and the appropriate speed was set. Due to the difference in methanol concentrations on both sides, the osmotic pressure would cause methanol to permeate towards the side with deionized water. The refractive index of the solution in Chamber B was measured every 30 min. The molar concentration of methanol in Chamber B at each time point was calculated using Formula (5). The concentration-time curve of the methanol solution in Chamber B was calculated and the slope S was obtained by linear fitting. The methanol permeation rate of the PEM in the wet state was calculated according to Formula (6).

(6) $$P=\frac{SV_{B}L}{AC_{A}}$$

where *V_B_* is the volume of the solution in Chamber B (cm^3^), *L* is the thickness of the membrane material sample (cm), *A* is the effective area of the membrane where methanol permeation occurs (cm^2^), and *C_A_* is the molar concentration of the methanol solution in chamber *A* (mol/L).

#### 2.6.9. Measurement of Oxidation Stability

In this study, Fenton’s reagent was used to test the antioxidant properties of the membrane. The antioxidant properties of the PEMs were evaluated based on the mass loss of the membrane in Fenton’s reagent. First, a certain amount of Fenton’s reagent (2 mg/L FeSO_4_ dissolved in 3% H_2_O_2_ solution) was weighed, and the primary SPEEK membrane, SPEEK/MXene composite membrane and SPEEK/MXene/Pt-TiO_2_ composite membranes with different contents were cut into fixed sizes. The membrane was placed in a volumetric flask containing 20 mL of Fenton’s reagent and then placed in an oven at 60 °C. The antioxidant properties of PEMs were determined based on the mass change ratio of the membrane within 6 h.

## 3. Results

### 3.1. Structure and Performance of Modified Pt-TiO_2_, f-MXene and SPEEK

#### 3.1.1. Analysis of Modified Pt-TiO_2_

**Figure 1:**
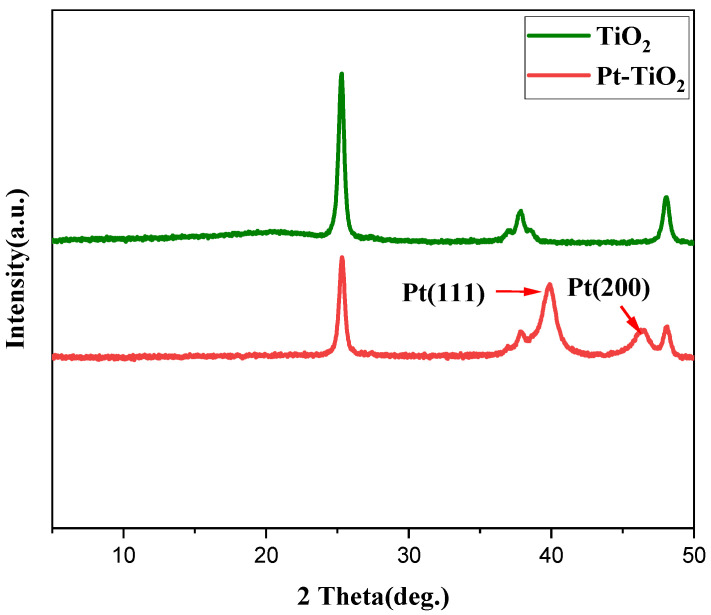
XRD patterns of TiO_2_ and modified Pt-TiO_2_ nanoparticles.

XRD patterns of TiO_2_ and modified Pt-TiO_2_ nanoparticles are shown in Figure 1. The characteristic diffraction peaks of TiO_2_ at 2θ = 25°, 37.5° and 47.9°are in accordance with the (101), (004) and (200) crystal planes of anatase TiO_2_, indicating that TiO_2_ is the anatase crystalline phase. However, the characteristic diffraction peaks of the modified Pt-TiO_2_ nanoparticles at 2θ = 39.8° and 46.2° are in accordance with the (111) and (200) crystal planes of Pt-TiO_2_, indicating that Pt metal has been loaded successfully onto the surface of TiO_2_.

Although there is a weaker tendency for the peaks of modified Pt-TiO_2_ compared with that of the TiO_2_, the characteristic peak of the modified Pt-TiO_2_ at 2θ = 25° corresponding to the anatase phase is still obvious, suggesting that the loading of Pt metal does not induce structural changes in TiO_2_.

**Figure 2:**
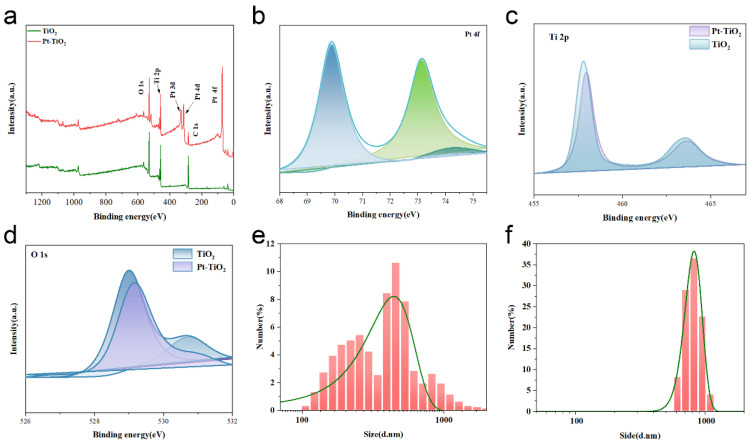
XPS measurement spectra of TiO_2_ and Pt-TiO_2_: (**a**) full spectrum of TiO_2_ and Pt-TiO_2_; (**b**) fine spectrum of Pt element in Pt-TiO_2_; (**c**) fine spectrum of Ti 2p in TiO_2_ and Pt-TiO_2_; (**d**) fine spectrum of O 1s in TiO_2_ and Pt-TiO_2_. Particle size distribution of TiO_2_ (**e**) and Pt-TiO_2_ (**f**).

Figure 2a–d show the XPS spectra of TiO_2_ and modified Pt-TiO_2_. It can be seen that the characteristic peaks of TiO_2_ and modified Pt-TiO_2_ appear at binding energies of 284.8, 460.2 and 531.1 eV, corresponding to C 1s, Ti 2p and O 1s, respectively.

Compared with the spectrum of TiO_2_, the characteristic peaks of Pt at binding energies 70.8, 313.1 and 330.1 eV are observed for the spectrum of Pt-TiO_2_, indicating further that Pt metal has been loaded successfully onto the surface of TiO_2_.

In the high-resolution Pt 4f level spectrum analysis of Pt-TiO_2_, the Pt 4f-level region predominantly exhibits two peaks resulting from the spin-orbit split peaks of Pt (4f_7/2_) and Pt (4f_5/2_) with binding energies of 70.2 and 73.1 eV, respectively. The peak at 70 eV binding energy corresponds to Pt (0), the peak at 73.1 eV corresponds to Pt (2^+^), and the peak at 74.5 eV corresponds to Pt (4^+^) [26]. The results show that there are three forms of Pt deposited on the TiO_2_ surface. A minor occurrence of Pt metal particles is oxidized into Pt^2+^ during the preparation of Pt-TiO_2_, indicating that Pt on the surface of TiO_2_ nanoparticles predominantly exists in the form of metallic Pt and Pt oxide [27].

The particle size distributions of TiO_2_ and Pt-TiO_2_ nanoparticles are shown in Figure 2e,f. Compared with TiO_2_, the Pt-TiO_2_ nanoparticles modified with the silane coupling agent show a more uniform particle size and a narrower size distribution, indicating that KH-550 modification effectively improves the dispersion of nanoparticles, which is conducive to their uniform dispersion in the SPEEK matrix.

#### 3.1.2. Analysis of SPEEK

**Figure 3:**
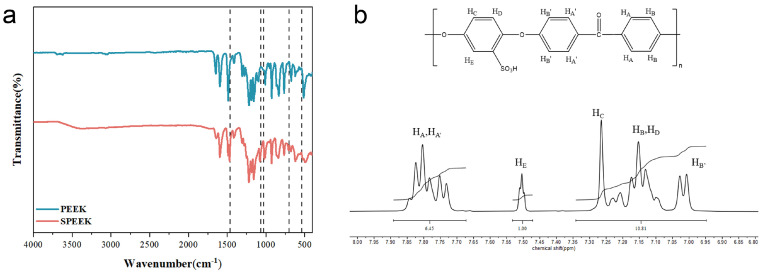
(**a**) FTIR image of PEEK and SPEEK. (**b**) ^1^HNMR spectrum of SPEEK in DMSO-d6.

Figure 3a shows the FTIR spectra of PEEK and SPEEK. Compared with PEEK, SPEEK exhibits new characteristic absorption peaks at 530 cm^−1^, 680 cm^−1^, 1025 cm^−1^, 1078 cm^−1^ and 1480 cm^−1^. The peaks at 530 cm^−1^, 680 cm^−1^ and 1078 cm^−1^ correspond to the stretching vibrations of O=S=O, indicating that sulfonic acid groups are grafted successfully onto the PEEK structure. The peak of PEEK and SPEEK at 1650 cm^−1^ represents the carbonyl (C=O) characteristic peak. Compared with PEEK, the FTIR spectrum of SPEEK shows that the C-C characteristic peak of aromatic compounds at 1490 cm^−1^ splits into two peaks. The emergence of a new absorption peak is attributed to the substitution reaction of sulfonic acid groups [28].

The DS of SPEEK is determined by nuclear magnetic resonance spectrometer. Figure 3b shows the ^1^HNMR spectrum of the SPEEK polymer. By substituting the result of the integrated area into Formula (7) [29], it can be seen that the DS of the synthesized SPEEK is 62.3%. This *DS* value is within the optimal range (approximately 60%) reported by Song et al. [30], which balances proton conductivity and mechanical durability without excessive swelling.

(7) $$\frac{DS}{12-2DS}=\frac{A_{H_{E}}}{\sum A_{H_{A,A^{\prime },B,B^{\prime },C,D}}}$$

Among them, $A_{H_{E}}$ is the integral of *H_E_*, and $\sum A_{H_{A,A^{\prime },B,B^{\prime },C,D}}$ is the sum of the integrals of *H_A_*, *H*_*A*′_, *H_B_*, *H*_*B*′_, *H_C_* and *H_D_*.

#### 3.1.3. Analysis of f-MXene

**Figure 4:**
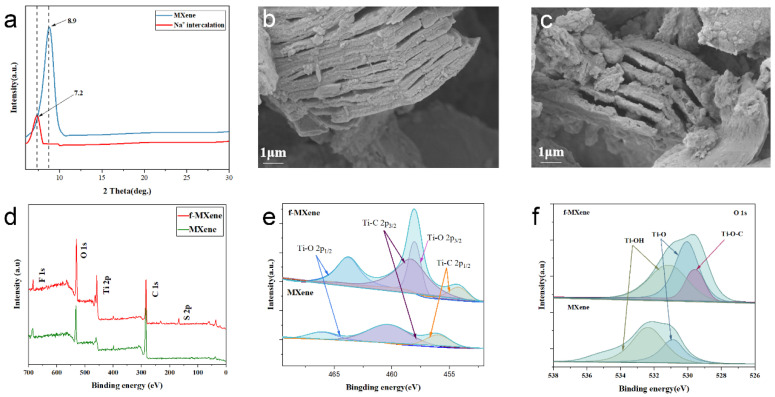
(**a**) XRD of MXene and Na^+^ intercalation MXene nanoparticles. SEM of MXene (**b**) and Na^+^ intercalation MXene (**c**) nanoparticles. XPS measurement spectra of MXene and f-MXene: (**d**) full spectrum; (**e**) fine spectrum of Ti 2p; (**f**) fine spectrum of O 1s.

The XRD spectra of MXene and f-MXene are shown in Figure 4a. It can be seen that the (002) peak of MXene shifts significantly downwards from 8.9° to 7.2° after treatment with NaOH solution. It is attributed to the introduction of Na^+^ into the gaps between the MXene layers, resulting in an increased interlayer spacing of MXene. In turn, it causes the (002) peak of MXene to shift downwards. This result is consistent with the literature reports that the interlayer spacing of MXene increases after alkalization, resulting in the MXene (002) peak shifting to a smaller angle [31], indicating that MXene is functionalized successfully.

Figure 4b,c display the morphological images of MXene and f-MXene. From the SEM images, the structural composition of MXene is obtained by etching off the metal layers of Ti_3_AlC_2_. MXene has a multi-layered nanosheet structure with a large specific surface area, which can provide more transfer sites for subsequent chemical grafting reactions, thereby facilitating chemical modifications with inorganic fillers [31]. After alkali treatment, Na^+^ enters the interlayer spacing of MXene. As shown in the images, the intercalation of Na^+^ causes the expansion of the interlayer spacing of MXene, which is beneficial for the subsequent modification of the functional groups on the MXene surface. In summary, the increased interlayer spacing further confirms the successful preparation of f-MXene.

Figure 4d–f present the wide-scan spectra of MXene and f-MXene, as well as the high-resolution scan spectra of Ti 2p and O 1s. The XPS analysis spectra can clearly describe the elemental composition of compounds. The wide-scan spectra reveal characteristic peaks of F 1s (684.08 eV), O 1s (530.08 eV), Ti 2p (458.08 eV) and C 1s (283.08 eV) in both materials. Notably, the wide-scan spectrum of f-MXene shows the appearance of the S 2p peak (167.08 eV) [32], indicating the introduction of sulfonic acid groups onto the surface of MXene. Compared with unmodified MXene, the binding energy in the Ti 2p region has increased from 455 eV to 456 eV after the introduction of sulfonic acid groups, suggesting a decrease in the electron density of Ti atoms due to the diazotization reaction. This phenomenon is similar to that reported in the literature [33], where the diazotization reaction involves interactions between TiNx and titanium nitride.

After the diazotization reaction, the electron cloud density around Ti atoms decreases, promoting the adsorption of -OH groups and driving electron transfer in a positive direction. High-resolution scans of the O 1s region for unmodified MXene and f-MXene reveal an increase in oxygen content in the f-MXene, indicating the successful grafting of sulfonic acid groups. The full spectra analysis also provides insights into the chemical bond compositions within MXene. According to the O 1s high-resolution spectrum, MXene and f-MXene have characteristic peaks representing Ti-OH at 532.3 eV and 531.5 eV, respectively. At the same time, MXene and f-MXene have characteristic peaks representing Ti-O at 531 eV and 530.5 eV, respectively. Notably, f-MXene exhibits a unique peak at 529.6 eV in the O 1s high-resolution spectrum, while the unmodified MXene lacks this peak. According to the literature, this peak corresponds to the binding energy of O 1s in the metal-O-C bond [34]. Furthermore, the O 1s energy spectrum of sulfonic acid groups also aligns with this binding energy position, indicating the presence of metal-O-C chemical bonds, where the benzene sulfonic acid groups are grafted onto the surface of MXene nanolayers.

### 3.2. Performance of the Composite Membranes

#### 3.2.1. FTIR Analysis of Composite Membranes

**Figure 5:**
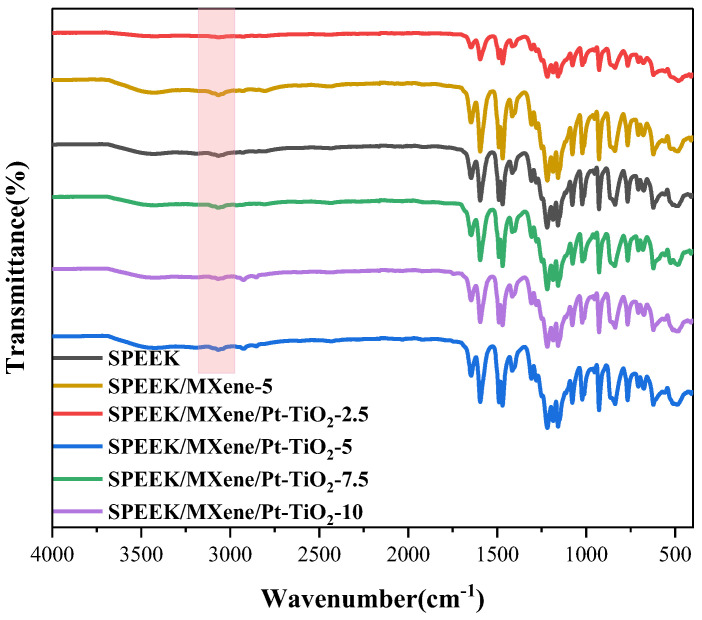
FTIR spectra of primary SPEEK, SPEEK/MXene-5 and SPEEK/MXene/Pt-TiO_2_ composite membranes with different Pt-TiO_2_ contents.

Figure 5 shows the FTIR spectra of the primary SPEEK membrane, SPEEK/MXene-5 membrane and SPEEK/MXene/Pt-TiO_2_ composite membranes with different Pt-TiO_2_ contents. Compared with the primary SPEEK membrane, all composite membranes exhibit a new broad absorption band at approximately 3100 cm^−1^, attributed to the O-H stretching vibrations of hydroxyl groups on the surfaces of Pt-TiO_2_ and f-MXene. No new characteristic peaks corresponding to chemical bond formation are observed in other regions, indicating that the inorganic nanofillers interact with the SPEEK matrix primarily through physical interfacial interactions rather than chemical reactions. Notably, the characteristic peak intensities of the SPEEK/MXene-5 membrane at 1180–1200 cm^−1^ (asymmetric S=O stretching) and 1030–1080 cm^−1^ (symmetric S=O stretching) are significantly enhanced compared with those of the primary SPEEK membrane. This enhancement directly confirms the successful grafting of sulfonic acid groups onto the MXene surface, thereby introducing additional proton exchange sites into the membrane matrix.

#### 3.2.2. Proton Conductivity

**Figure 6:**
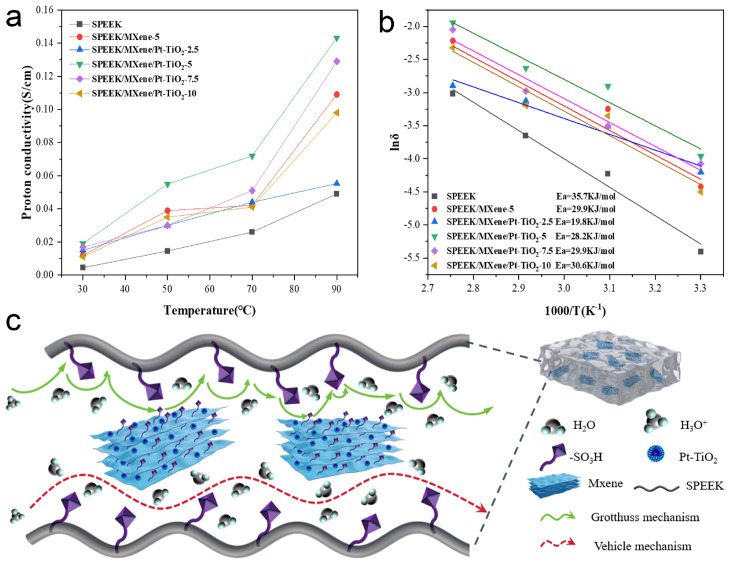
(**a**) Proton conductivity, (**b**) Arrhenius plot of SPEEK membrane, SPEEK/MXene-5 composite membrane and SPEEK/MXene/Pt-TiO_2_ composite membranes; (**c**) proton conduction mechanism diagram of SPEEK/MXene/Pt-TiO_2_ composite membranes.

High proton conductivity is an essential property of a PEM for evaluating the performance of FCs. Proton conductivities of the primary SPEEK membrane, SPEEK/MXene-5 composite membrane and SPEEK/MXene/Pt-TiO_2_ composite membranes incorporated into different nanofillers and contents at different temperatures (100% RH humidity) are shown in Figure 6a. Proton conductivity of SPEEK/MXene/Pt-TiO_2_-x composite membranes is obviously higher than those of primary SPEEK membrane and SPEEK/MXene-5 composite membrane. In addition, at 90 °C and 100% RH, the SPEEK/MXene/Pt-TiO_2_-5 composite membrane containing 5% f-MXene and 5% Pt-TiO_2_ exhibits a high protonic conductivity of 0.143 S/cm, which is higher than the proton conductivity of Nafion composite membranes at similar test conditions (0.06–0.13 S/cm) [35]. This represents increases of about 192% and about 31% compared with the primary SPEEK membrane and the SPEEK/MXene-5 composite membrane under the same conditions, respectively. This value also compares favorably with other SPEEK-based composite membranes reported in the literature; for instance, Ling et al. reported a proton conductivity of 139.2 mS cm^−1^ at 90 °C and 98% RH for a SPEEK/IL@HNTs membrane [36], which is slightly lower than the value obtained in this work.

As shown in Figure 6a, the proton conductivity of SPEEK/MXene/Pt-TiO_2_-x composite membranes increases gradually with an increase in the test temperature. This is attributed to the increased diffusion rate of water molecules at higher temperatures, making the movement of polymer macromolecular chains relatively easier and facilitating the formation of hydrophilic regions within the composite membrane, thereby promoting proton transport.

To substantiate the synergistic effect between the two fillers, control experiments using single-filler systems (SPEEK/MXene with varying f-MXene contents) were conducted, with the corresponding proton conductivity and activation energy data summarized in Table 1. At 90 °C and 100% RH, the SPEEK/MXene-5 membrane exhibits a proton conductivity of 0.109 S/cm, which is significantly lower than that of the dual-filler SPEEK/MXene/Pt-TiO_2_-5 membrane (0.143 S/cm) under the same conditions, clearly demonstrating a pronounced synergistic enhancement beyond the individual contributions of each filler. The increase in proton conductivity of the dual-filler system can be attributed to the synergistic optimization of membrane microstructure and proton transport pathways. Specifically, f-MXene provides a high density of sulfonic acid groups, which increases the number of proton exchange sites and reduces the distance between them, thereby facilitating the Grotthuss (hopping) mechanism. Similarly, the sulfonation modification strategy of MXene in this study has a comparable surface engineering approach to the amino-functionalization of carbon nanotubes used by Bai et al. [18]. Meanwhile, the hydrophilic Pt-TiO_2_ nanoparticles enhance water retention, promoting the formation of continuous hydrophilic domains and supporting the vehicle mechanism by maintaining a high local water content for proton diffusion. Critically, the hydrogen bonding interface interaction between f-MXene and Pt-TiO_2_ not only improves the dispersion of both nanofillers within the SPEEK matrix, preventing severe aggregation, but also creates an interconnected network of proton-conductive pathways. This well-dispersed state allows the efficient integration of the hopping-dominated conduction from f-MXene and the water-assisted transport from Pt-TiO_2_, enabling both Grotthuss and vehicle mechanisms to operate in a cooperative manner. This performance improvement mechanism is similar to the interfacial proton hopping effect in the WO_3_/SPEEK system reported by Sun et al. [15].

However, with an increase in the content of Pt-TiO_2_ nanoparticles, a slight decrease in proton conductivity of the composite membrane is observed, although it remains significantly higher than that of the primary SPEEK membrane. This decrease can be attributed to the aggregation of excess Pt-TiO_2_ nanoparticles within the polymer matrix, weakening the hydrogen bonding interaction between Pt-TiO_2_ and MXene, occupying the free volume within the polymer matrix, reducing macromolecular chain mobility, blocking proton transport channels and consequently impeding proton transport. In conclusion, the appropriate incorporation of f-MXene and modified Pt-TiO_2_ nanofillers can effectively enhance the proton conductivity of the PEMs.

In order to understand the mechanism of proton transport in composite PEMs, the activation energy values for proton conduction in pristine SPEEK membrane and all composite membranes are calculated according to the Arrhenius equation (shown in Formula (8)) and proton conductivity values. The activation energy represents the energy required for proton transport through the membrane. Here, σ represents the proton conductivity, σ_0_ is the pre-exponential factor, *T* is the absolute temperature, and *R* is the gas constant.

(8) $$\sigma =\frac{\sigma_{0}}{T}\times e^{-\frac{Ea}{RT}}$$

Arrhenius plots for primary SPEEK membrane, SPEEK/MXene composite membrane and SPEEK/MXene/Pt-TiO_2_ composite membranes are presented in Figure 6b. Compared with that of the primary SPEEK membrane, the activation energy of SPEEK/MXene/Pt-TiO_2_ composite membranes containing f-MXene and Pt-TiO_2_ inorganic nanofillers decreases significantly. Specifically, the activation energy of the primary SPEEK membrane is 35.7 kJ/mol, that of the SPEEK/MXene composite membrane is 29.9 kJ/mol, and the values for the SPEEK/MXene/Pt-TiO_2_ composite membranes are 19.8, 28.2, 29.9 and 30.6 kJ/mol, respectively, implying that the composite membranes have higher proton conductivity than primary SPEEK membrane. Activation energy values derived from Arrhenius plots were in the range of 19.8–35.7 kJ/mol and indicate that proton conduction of the composite membranes mostly follows the Grotthuss mechanism because the activation energy for proton transportation of the Grotthuss mechanism is in the range of 14–40 kJ/mol [37,38]. Proton conduction mechanism diagram of SPEEK/MXene/Pt-TiO_2_ composite membranes is shown in Figure 6c. Therefore, both the Grotthuss mechanism and the vehicle mechanism can be accepted for proton conduction across the composite membranes under the synergic action of f-MXene, providing a higher number of exchangeable proton sites, while modified TiO_2_ enhances water retention.

#### 3.2.3. WU, SR and Mechanical Properties

**Figure 7:**
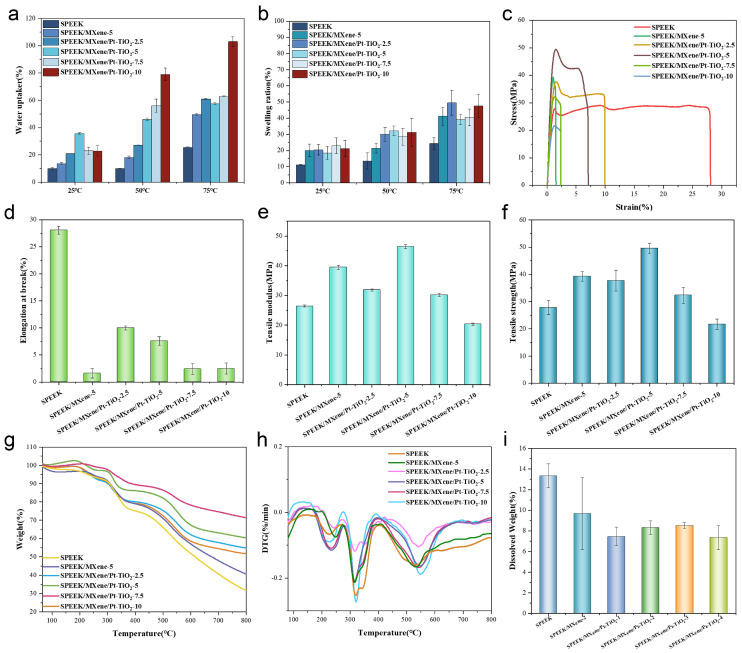
WU (**a**) and SR (**b**) of the primary SPEEK membrane, the SPEEK/MXene composite membrane and the SPEEK/MXene/Pt-TiO_2_ composite membrane at different temperatures. Mechanical properties of the primary SPEEK, the SPEEK/MXene composite membrane and the SPEEK/MXene/Pt-TiO_2_ composite PEM: (**c**) stress–strain curve; (**d**) elongation at break; (**e**) tensile modulus; (**f**) tensile strength. TG curves (**g**), DTG curves (**h**) and (**i**) oxidation stability curves of the primary SPEEK membrane, the SPEEK/MXene composite membrane and the SPEEK/MXene/Pt-TiO_2_ composite membrane.

The WU of the primary SPEEK membrane, SPEEK/MXene composite membrane and SPEEK/MXene/Pt-TiO_2_ composite membranes at different temperatures is shown in Figure 7a. The WU values of the SPEEK/MXene/Pt-TiO_2_ composite membranes gradually increase with an increase in modified Pt-TiO_2_ contents and elevated testing temperatures. For example, the WU of the SPEEK/MXene/Pt-TiO_2_-10 composite PEM at 75 °C can be up to 103%, which represents an increase of about 53% compared with that of the SPEEK/MXene PEM. This increase is attributed to the large number of hydrophilic -OH groups attached onto the surface of modified Pt-TiO_2_ nanoparticles, which form strong hydrogen bonds and interactions with water molecules. Additionally, the layered structure of f-MXene and the hydrogen bonding between Pt-TiO_2_ and MXene can stimulate Pt-TiO_2_ nanoparticles to enter the f-MXene interlayer, resulting in a significant increase in the WU values for the composite membrane. Furthermore, because Pt-TiO_2_ nanoparticles are distributed into the f-MXene interlayer, water molecules within the MXene interlayer spacing do not diffuse easily, enhancing the water retention capacity of the composite membranes.

SR determines the dimensional stability of PEMs during their usage. An excessively high SR causes the PEMs to deform easily, thereby affecting their performance. The SR of the primary SPEEK membrane, the SPEEK/MXene composite membrane and the SPEEK/MXene/Pt-TiO_2_ composite membranes at different temperatures is shown in Figure 7b. It can be observed that the swelling behavior of the SPEEK/MXene composite membranes increases after incorporating f-MXene. This is because a large number of hydroxyl and sulfonic acid groups are present on the surface of MXene, generating hydrophilic regions in the membrane and resulting in the membrane absorbing water and thus swelling. However, a slight decrease in SR for the SPEEK/MXene composite membranes is observed with the introduction of modified Pt-TiO_2_ because Pt-TiO_2_ nanoparticles exist within the interlayer spacing of MXene. The hydrogen bonding interaction among the oxygen-containing groups on the surfaces of MXene, Pt-TiO_2_ nanoparticles, and the SPEEK matrix can result in forming a more compact membrane structure, enhancing dimensional stability and causing a slight decrease in SR with increasing WU.

The mechanical properties of PEM seriously affect the manufacturing conditions of FCs and their durability. The mechanical properties of the primary SPEEK membrane, the SPEEK/MXene composite membrane and the SPEEK/MXene/Pt-TiO_2_ composite membranes are shown in Figure 7c–f. The tensile strength values of the primary SPEEK, SPEEK/MXene-5, and SPEEK/MXene/Pt-TiO_2_ membranes with varying filler contents were 27.79, 39.30, 37.73, 49.50, 32.30 and 21.73 MPa, respectively. Among all membranes, SPEEK/MXene/Pt-TiO_2_-5 exhibited the highest tensile strength. Compared with the primary SPEEK membrane and the SPEEK/MXene composite membrane, the tensile strength of the SPEEK/MXene/Pt-TiO_2_-5 membrane has increased by 78.12% and 25.95%, respectively. The tensile curves indicate that the incorporation of an appropriate amount of inorganic nanofillers can enhance the tensile strength of the composite membranes and improve their mechanical properties. This enhancement can be attributed to two main factors. First, the high aspect ratio of MXene facilitates efficient stress transfer and forms a reinforcing network within the membrane. Second, the abundant hydroxyl groups on Pt-TiO_2_ form hydrogen bonds with the sulfonic acid groups in the SPEEK matrix. These interactions collectively restrict polymer chain mobility under deformation, resulting in a more compact membrane structure [39]. In addition, compared with the SPEEK/MXene composite membrane, the SPEEK/MXene/Pt-TiO_2_ composite membrane showed improved elongation at break, indicating enhanced ductility, which is critical for both the durability and assembly of PEMFC components.

#### 3.2.4. Thermal Stability, Oxidation Stability and Cross-Section Morphology

PEMs should have good thermal stability to ensure the durability of fuel cells under high-temperature and high-humidity conditions. The TG and DTG curves of the primary SPEEK membrane, the SPEEK/MXene composite membrane and the SPEEK/MXene/Pt-TiO_2_ composite membranes are depicted in Figure 7g,h. Both the primary SPEEK membrane and the composite membranes exhibit weight loss behavior below 220 °C due to the volatilization of free water, bound water and residual solvents. The second weight loss stage occurs in the range of 220–500 °C, corresponding to the decomposition of sulfonic acid groups and hydroxyl groups derived from the SPEEK side chains and the surface functional groups of the inorganic fillers. The weight loss between 500 and 600 °C is attributed to the degradation of the SPEEK main chain. The decomposition process of the composite membranes is generally consistent with that of the primary SPEEK membrane; however, a notable difference is observed in the maximum decomposition rate temperature during the main-chain degradation stage. As shown in the DTG curves, the primary SPEEK membrane exhibits a maximum decomposition rate temperature of 520 °C, while that of the SPEEK/MXene/Pt-TiO_2_ composite membrane increases to approximately 550 °C, with the SPEEK/MXene composite membrane lying in between. This indicates that the introduction of inorganic nanofillers, particularly the further incorporation of Pt-TiO_2_, effectively enhances the thermal stability of the composite membranes. In addition, the TG curves show that both composite membranes exhibit higher residual weights at 800 °C compared with the primary SPEEK membrane, further confirming the improved thermal stability upon the addition of inorganic nanofillers.

Membrane oxidative stability, which is evaluated by immersing the dried membranes in Fenton’s reagent, is a key parameter for PEMFCs to obtain high durability. MXene was selected in this study because its hydrophilic terminal groups and high specific surface area can provide additional proton transport pathways and enhance interfacial compatibility with the SPEEK matrix, which are highly desirable for improving proton conductivity. However, pristine MXene is known to have limited oxidation stability in aqueous environments. To address this limitation, the SPEEK matrix physically encapsulates the MXene nanosheets, isolating them from direct contact with oxidative species. Additionally, f-MXene with grafted sulfonic acid groups exhibits improved interfacial compatibility with the SPEEK matrix, resulting in a more compact composite structure that further restricts the permeation of oxidative radicals. Figure 7i shows that the oxidative stability of the composite membranes can be enhanced by incorporating f-MXene nanofillers and further improved by the introduction of Pt-TiO_2_. The SPEEK/MXene/Pt-TiO_2_-5 composite membrane has outstanding oxidative stability, which is attributed to an appropriate amount of modified Pt-TiO_2_ nanoparticles incorporated into the interlayer spaces of f-MXene. However, excessive Pt-TiO_2_ nanoparticles may aggregate in the polymer matrix and hinder the movement of polymer chains, resulting in a decrease in oxidation stability for the SPEEK/MXene/Pt-TiO_2_-x composite membrane.

**Figure 8:**
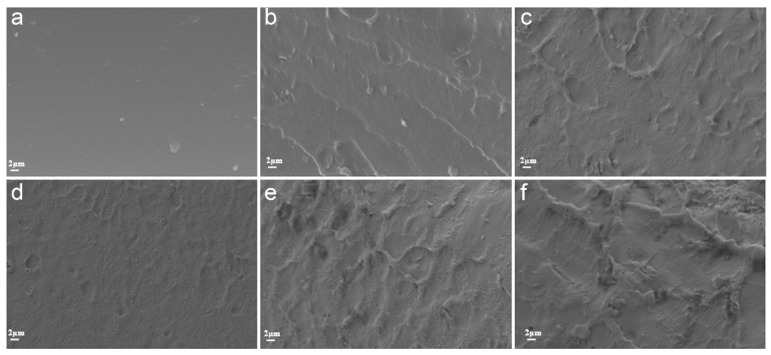
SEM images of the primary SPEEK membrane and composite membranes: (**a**) SPEEK; (**b**) SPEEK/MXene-5; (**c**) SPEEK/MXene/Pt-TiO_2_-2.5; (**d**) SPEEK/MXene/Pt-TiO_2_-5; (**e**) SPEEK/MXene/Pt-TiO_2_-7.5; (**f**) SPEEK/MXene/Pt-TiO_2_-10.

The cross-section morphologies of the primary SPEEK membrane, the SPEEK/MXene composite membrane and the SPEEK/MXene/Pt-TiO_2_ composite membranes are depicted in Figure 8. The surface morphology of the primary SPEEK membrane is relatively smooth. However, the surface morphology of the SPEEK/MXene composite membrane exhibits uniform texture, which is attributed to the uniform distribution of the layered f-MXene nanofillers incorporating into the SPEEK matrix. In addition, with the addition of Pt-TiO_2_ nanoparticles, the cross-section morphology of the composite membrane also has a relatively dense structure with no defects when modified Pt-TiO_2_ nanoparticles are incorporated into the SPEEK/MXene membrane. Compared with other SPEEK/MXene/Pt-TiO_2_-x composite membranes, the SPEEK/MXene/Pt-TiO_2_-5 composite membrane exhibits better morphology and fewer defects, which might help to create proton-conducting channels and improve some properties of the composite membranes.

#### 3.2.5. IEC, Methanol Permeability and Selectivity

The IEC values of all the membranes are summarized in Table 2. The IEC values of the SPEEK/MXene composite membranes are higher than that of the primary SPEEK membrane. This is attributed to the abundant hydroxyl and sulfonic acid groups attached to the surface of incorporated f-MXene, effectively increasing the proton exchange capacity. However, the IEC values of the SPEEK/MXene/Pt-TiO_2_ composite membranes do not show a substantial increase upon incorporation of modified Pt-TiO_2_ nanoparticles and decrease with an increase in the Pt-TiO_2_ content. Therefore, incorporating a moderate content of modified Pt-TiO_2_ containing hydrophilic groups is beneficial to the adsorption of water molecules, which makes it easier for Na^+^ to replace H^+^ within the composite membrane, thereby increasing the IEC [40].

Methanol permeability is an essential parameter that affects the performance of PEMFCs. PEM materials with the lowest possible methanol permeability are generally desired. It can be seen from Table 2 that the methanol permeability of the SPEEK/MXene composite membranes is lower than that of the primary SPEEK membrane. This could be due to the hydrogen bonding interactions between the oxygen-containing groups, sulfonic acid groups and fluorine functional groups on the surface of f-MXene and the sulfonic acid groups in the SPEEK matrix. These interactions promote a tighter arrangement between polymer chains, thereby reducing the methanol permeability of the composite membranes. Additionally, the methanol permeability of the SPEEK/MXene/Pt-TiO_2_-5 composite membrane is the lowest (2.27 × 10^−7^ cm^2^/s) among all the membranes. This value is significantly lower than that of other sulfonated aromatic polymer-based composite membranes reported recently, where methanol permeability values on the order of 10^−6^ cm^2^/s are commonly observed [11]. The optimal methanol permeability at 5 wt.% Pt-TiO_2_ loading is ascribed to the fillers occupying interlayer voids and strengthening interfacial interactions, leading to a more compact membrane structure. However, further increase in the Pt-TiO_2_ content leads to filler aggregation, which disrupts the uniform dispersion of the nanofillers within the SPEEK matrix, creating additional pathways for methanol transport and consequently increasing methanol permeability.

The selectivity values of all the composite membranes are also listed in Table 2. It can be seen that the incorporated MXene and Pt-TiO_2_ nanoparticles can significantly improve the selectivity of the composite PEMs. The selectivity value of the SPEEK/MXene/Pt-TiO_2_-5 composite membrane is about two times greater than that of the SPEEK/MXene composite membrane. This could be because the hydrogen bonding interactions between the oxygen groups on the Pt-TiO_2_ surface and the MXene layer can result in a more uniform dispersion of the inorganic nanofillers in the SPEEK polymer matrix. Additionally, the incorporation of Pt-TiO_2_ enhances the membrane’s self-wettability, and the increased water content improves the overall performance of the composite membranes.

## 4. Conclusions

SPEEK-based composite PEMs have become one of the research hotspots in recent years due to their good thermal stability, chemical stability and low cost. However, inorganic nanofillers have poor compatibility with the SPEEK polymer matrix and are prone to agglomeration, which limits their applications in fuel cells. In this study, we developed composite membranes by incorporating functionalized MXene and modified Pt-TiO_2_ nanoparticles through a solution casting technique for PEM in fuel cell applications. The results show that the SPEEK/MXene/Pt-TiO_2_ composite membrane has excellent comprehensive performance, without sacrificing other properties, when the Pt-TiO_2_ content is 5%. Compared with the primary SPEEK membrane and the SPEEK/MXene composite membrane, the proton conductivity (0.143 S·cm^−1^) of the SPEEK/MXene/Pt-TiO_2_-5 composite membrane increased by 192% and 31%, the selectivity coefficient (8.37) was 8.05 times and 2.08 times higher, the tensile strength (49.50 MPa) increased by 78.12% and 25.95%, and the methanol permeability (2.27 ± 0.02 × 10^−7^ cm^2^·s^−1^) decreased by 47.3% and 23.8%, respectively. The SPEEK/MXene/Pt-TiO_2_ composite membranes show self-humidifying properties and super-protonic conductivity, which is higher than the proton conductivity of Nafion composite membranes under similar test conditions. It should be emphasized that all performance results presented herein are based on initial characterizations, and long-term cycling stability and durability tests have not been carried out in this work, which is a limitation of the present study. Therefore, the SPEEK-based composite membranes may potentially find application as PEM materials for fuel cells, and further investigations into their long-term operational reliability will be conducted in future work.

## Figures and Tables

**Table 1:** Proton conductivity (σ) at 90 °C and 100% RH and activation energy (Ea) of SPEEK composite membranes with various f-MXene contents.

Sample	σ (S/cm)	Ea (kJ/mol)
SPEEK/MXene-2.5	0.061	37.4
SPEEK/MXene-5	0.109	29.9
SPEEK/MXene-7.5	0.067	31.5
SPEEK/MXene-10	0.065	30.6

These data are provided as a control to assess the individual effect of f-MXene fillers, enabling comparison with the dual-filler system (f-MXene/Pt-TiO_2_) to clarify the synergistic mechanism in proton conduction.

**Table 2:** IEC, methanol permeability and selectivity of the primary SPEEK membrane, the SPEEK/MXene composite membrane and the SPEEK/MXene/Pt-TiO_2_ composite membranes with different contents.

Sample	IEC (mmol/g)	Methanol Permeability (P, ×10^−7^ cm^2^·s^−1^)	Selectivity (SP, ×10^4^ S·s·cm^−3^)
SPEEK	1.50 ± 0.06	4.31 ± 0.23	1.04
SPEEK/MXene-5	3.73 ± 0.08	2.98 ± 0.04	4.02
SPEEK/MXene/Pt-TiO_2_-2.5	3.89 ± 0.03	3.19 ± 0.05	4.70
SPEEK/MXene/Pt-TiO_2_-5	3.76 ± 0.12	2.27 ± 0.02	8.37
SPEEK/MXene/Pt-TiO_2_-7.5	2.79 ± 0.09	2.83 ± 0.13	6.01
SPEEK/MXene/Pt-TiO_2_-10	2.54 ± 0.05	2.71 ± 0.17	4.09

## Data Availability

The data are available from the corresponding author on reasonable request.
